# The Role of DNA Methylation and Histone Modifications in Neurodegenerative Diseases: A Systematic Review

**DOI:** 10.1371/journal.pone.0167201

**Published:** 2016-12-14

**Authors:** Ke-xin Wen, Jelena Miliç, Bassem El-Khodor, Klodian Dhana, Jana Nano, Tammy Pulido, Bledar Kraja, Asija Zaciragic, Wichor M. Bramer, John Troup, Rajiv Chowdhury, M. Arfam Ikram, Abbas Dehghan, Taulant Muka, Oscar H. Franco

**Affiliations:** 1 Department of Epidemiology, Erasmus MC, Rotterdam, the Netherlands; 2 Research and Development, Metagenics, Inc, United States of America; 3 Department of Biomedical Sciences, Faculty of Medicine, University of Medicine, Tirana, Albania; 4 University Clinic of Gastrohepatology, University Hospital Center Mother Teresa, Tirana, Albania; 5 Medical Library, Erasmus MC, Rotterdam, The Netherlands; 6 Department of Public Health & Primary Care, Cardiovascular Epidemiology Unit, University of Cambridge, Cambridge, CB1 8RN, United Kingdom; University of Missouri Kansas City, UNITED STATES

## Abstract

**Importance:**

Epigenetic modifications of the genome, such as DNA methylation and histone modifications, have been reported to play a role in neurodegenerative diseases (ND) such as Alzheimer’s disease (AD) and Parkinson’s disease (PD).

**Objective:**

To systematically review studies investigating epigenetic marks in AD or PD.

**Methods:**

Eleven bibliographic databases (Embase.com, Medline (Ovid), Web-of-Science, Scopus, PubMed, Cinahl (EBSCOhost), Cochrane Central, ProQuest, Lilacs, Scielo and Google Scholar) were searched until July 11th 2016 to identify relevant articles. We included all randomized controlled trials, cohort, case-control and cross-sectional studies in humans that examined associations between epigenetic marks and ND. Two independent reviewers, with a third reviewer available for disagreements, performed the abstract and full text selection. Data was extracted using a pre-designed data collection form.

**Results:**

Of 6,927 searched references, 73 unique case-control studies met our inclusion criteria. Overall, 11,453 individuals were included in this systematic review (2,640 AD and 2,368 PD outcomes). There was no consistent association between global DNA methylation pattern and any ND. Studies reported epigenetic regulation of 31 genes (including cell communication, apoptosis, and neurogenesis genes in blood and brain tissue) in relation to AD and PD. Methylation at the *BDNF*, *SORBS3* and *APP* genes in AD were the most consistently reported associations. Methylation of α-synuclein gene (*SNCA*) was also found to be associated with PD. Seven studies reported histone protein alterations in AD and PD.

**Conclusion:**

Many studies have investigated epigenetics and ND. Further research should include larger cohort or longitudinal studies, in order to identify clinically significant epigenetic changes. Identifying relevant epigenetic changes could lead to interventional strategies in ND.

## Introduction

Alzheimer’s disease (AD) and Parkinson’s disease (PD) are the most common neurodegenerative disorders and are a major cause of disability and premature death among older people worldwide [1–3]. Due to global population ageing, prevalence of AD and PD is expected to increase, imposing a social and economic burden on society [4, 5]. The causes of most cases of neurodegenerative diseases remain largely unknown. However, in the last decade great advances have been made in our understanding of the pathogenetic mechanisms that lead to AD and PD [6–8]. It has been accepted that there are several genetic causes that play a role in the development of these disorders, including chromosome aberrations and gene mutations [8, 9]. Additionally, environmental exposures have been suggested to play a crucial role in the etiological process of neurodegenerative diseases. Both AD and PD are thought to be caused by complicated interactions between genetic and environmental factors [10]. Despite improvements in knowledge and understanding, there are currently no disease-modifying therapies for these diseases. A large amount of the variance in the risk of developing neurodegenerative diseases remains to be explained.

The epigenome is responsible for the molding and the three-dimensional structure of the genomic material in the cell nucleus. It provides a bridge between genes and environment and may help to improve our understanding on the etiology of complex diseases, AD, and PD [11]. Epigenetic mechanisms are known to alter gene expression or cellular phenotype in a heritable manner [12]. DNA methylation and modifications of histone proteins are the most intensively studied among the major epigenetic modifications. DNA methylation occurs when a methyl group is added at a cytosine nucleotide that precede guanines (so-called CpG dinucleotides). It further influences the function of DNA by activating or repressing the transcriptional activity of a gene [12]. Posttranslational histone modifications, such as methylation and acetylation of lysine residues on histone tails, are another type of epigenetic modification. Histone modifcations affect gene expression mainly by altering chromatin structure [12, 13].

Clinical features of neurological disorders and results from epidemiological studies suggest an epigenetic contribution to etiology of these diseases. Epigenetic modulation has been well documented in brain development, plastic changes, and in brain diseases including AD and PD. The most compelling evidence on the role of epigenetics on AD comes from the results of treatment of AD patients with inhibitors of histone deacetylases (HDAC). HDAC is a key enzyme involved in histone acetylation [14]. Also, in animal models of PD, HDAC inhibitor inhibits α-synuclein toxicity in the dopamine neuron, a common neuropathological feature of PD [15]. Dysregulation of DNA methylation in AD and PD patients is also well documented. Recent evidence shows that AD patients have an elevated DNA methylation state of repetitive elements [16]. Hypomethylation of the tumor necrosis factor (*TNF)* gene in cortex and higher levels of TNF-α cytokine in the cerebrospinal fluid has been reported in patients with PD [17]. TNF-α is one of the main proinflammatory cytokines that play a central role in the inflammatory response. TNF-α is also upregulated in AD patients and is involved in the pathogenesis of AD [18]. In dopaminergic regions of post-mortem brains, decreased methylation of the α-synuclein gene (*SNCA)* has been observed. The decreased methylation might be responsible for the accumulation of the protein α-synuclein, and thus the progression of PD [19, 20]. Moreover, DNA methylation and histone acetylation have recently been identified as playing a role in depression [21], an important feature of neurodegenerative diseases [22]. Emerging evidence shows that epigenetic mechanisms contribute to the process of learning and memory formation [23, 24]. Despite this evidence, to date, a comprehensive assessment of the role of epigenetic mechanisms in the development of AD and PD has not yet been done.

Therefore, we aimed to systematically review all available evidence in humans to assess the association of DNA methylation and histone modifications with the neurodegenerative disorders AD and PD.

## Materials and Methods

### Literature Search

This review was conducted using a predefined protocol in accordance with the PRISMA [25] and MOOSE [26] guidelines **(S1 File and S2 File)**. Eleven bibliographic databases (Embase.com, Medline (Ovid), Web-of-Science, Scopus, PubMed, Cinahl (EBSCOhost), Cochrane Central, ProQuest, Lilacs, Scielo and Google Scholar) were searched until July 11^th^ 2016 (date last searched) without any language restrictions, with the help of an experienced medical information specialist (WMB). The search strategy combined terms related to exposure (e.g., epigenetics, DNA methylation, histone, CpG) and outcomes (e.g., neurological disorders, dementia, Alzheimer, Parkinson). In databases where a thesaurus was available (Embase, Medline and Cinahl) articles were searched by thesaurus terms, title and/or abstract; other databases were searched only by title and/or abstract. We restricted the search to studies on human adults. The full search strategies of all databases are provided in **S3 File**. After eliminating duplications, we identified a total of 6927 potentially relevant citations. We retrieved reference lists of the studies and sought contact with experts to find further relevant publications.

### Study Selection and Inclusion Criteria

Included studies either described an association between epigenetic marks (global, site specific or genome-wide methylation of DNA) or histone modifications (methylation, phosphorylation, acetylation, ubiquitylation, and sumoylation) and neurodegenerative outcomes defined as AD and PD. There was no restriction based on the tissue type examined for epigenetic marks, and therefore, epigenetic marks assessed in any tissue (e.g. brain, blood) were included. We included cross-sectional, prospective, case-cohort and nested case control studies. Studies were excluded if they (i) examined epigenetic marks other than DNA methylation and histone modifications, such as noncoding RNAs; (ii) examined neurodegerative diseases other than AD and PD, such as Huntington’s disease, Prion disease, Motor neurone diseases, Spinocerebellar ataxia, Spinal muscular atrophy; (iii) were case studies or letters to the editor. Two independent reviewers (KW/JM and KD/JN/TP/BK/AZ) screened the retrieved titles and abstracts and selected eligible studies. In cases of disagreement, decision was made through consensus or consultation with a third independent reviewer (TM). Full texts were retrieved for studies that satisfied all selection criteria.

### Data Extraction

A predesigned data collection form was prepared to extract the relevant information from the selected studies, including study design, study population, location, age range, duration of follow up (for longitudinal studies), confounders, tissue sample, method used to assess epigenetic marks, type and numbers of neurodegenerative outcomes and reported measures of associations (e.g., correlation analysis, odds ratio, relative risks, confidence intervals). Two independent authors (KW and JM/TM) extracted the data.

### Assessing the risk of bias

Bias within each individual study was evaluated by two independent reviewers (KW and JM) using the validated Newcastle-Ottawa Scale, a semi-quantitative scale designed to evaluate the quality of nonrandomized studies [27]. The scores are provided in **S5 File**. Study quality was judged based on the selection criteria of participants, comparability of cases and controls, and exposure and outcome assessment. Studies that received a score of 9 stars were judged to be at low risk of bias; studies that scored 7 or 8 stars were considered to be at medium risk; those that scored 6 or less were considered to be at high risk of bias.

### Outcome Assessment

For each study, we defined whether an association was reported and whether direction and effect sizes were reported, when applicable.

## Results

**Fig 1:**
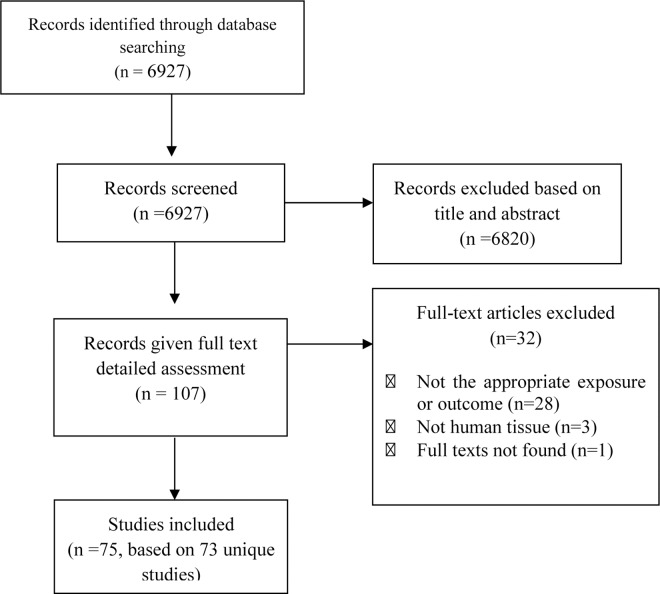
Flowchart of studies investigating epigenetic marks in relation to Alzheimer’s disease and Parkinson’s disease.

We identified 6927 potentially relevant publications (**Fig 1**) after removal of duplicate citations. Based on the title and abstracts, 107 articles were selected for detailed evaluation of their full texts. Of those, 32 articles were excluded for either having the wrong exposure or outcome (n = 28), reporting results from animal models (n = 3), or unavailable full texts (n = 1) (**Fig 1 and S4 File**). Seventy-five articles, based on 73 unique case-control studies, met our eligibility criteria and were included in this review.

### Summary of Included Studies

Overall, 11453 individuals were included within the systematic review, with a total of 2640 for AD and 2368 for PD outcomes. Of the 73 unique studies included, 13 studies assessed global DNA-methylation, 45 studies assessed DNA methylation in specific candidate genes, 8 studies used genome-wide approaches, 1 study assessed both global DNA methylation, histone modifications and DNA methylation in specific candidate genes, and 6 studies examined histone modifications in relation to ND (**Tables 1–3**). Twenty-nine studies assessed DNA methylation and/or histone modifications only in blood, 35 in the brain tissue, 8 studies in both blood and brain tissue and 1 study assessed methylation in skin fibroblasts. Fifty-seven studies examined AD as an outcome while 18 studies examined PD. Twenty-four studies included participants from USA, 11 studies from China, 4 studies included participants from more than 1 country and the rest included participants solely from Canada, Germany, United Kingdom, Italy, Spain, Japan, Sweden, Columbia, Australia, New Zealand, Serbia or Brazil (**Tables 1–3).** Three studies were judged at low risk of bias whereas the rest were at medium and high risk of bias (**S4 File**).

**Table 1:** Global DNA methylation in Alzheimer’s disease and Parkinson’s disease

Author	Population	No of cases	Tissue	Adjustment	Association	Comment
**ALZHEIMER’S DISEASE**						
**5mdC**						
Mastroeni D. et al, 2010[28]	USA, n = 40, 60–97 years, M and W	20	Human post-mortem brain tissue (neurons of entorhinal cortex layer II and other regions-cerrebellum)		Inverse association	Methylation levels were decreased in AD cases compared to controls (91.3% ± 1.3 in non-AD cases and 39.9% ± 3.4%, P<0.0001). No difference in methylation frequency in other regions of the brain such as the cerebellum.
Chouliaras L.et al, 2013[29]	USA, n = 20 and one pair of monozygotic twins discordant for AD), 76.64 ± 4.9 years, M and W	10	Hippocampal tissue	Age and gender	Inverse association	Decreased 5-mC and 5-hmC immunoreactivity in AD hippocampus (-19.6%, p = 0.006 and -20.2%, p = 0.012). Decreased level of 5-mC immunoreactivity in glial cells in the CA3 and CA1 region of the hippocampus (-26.9%, p = 0.016 and -25.7%, p = 0.003 respectively) as well as in the neurons of the CA1 region (-21.1%, p = 0.01). No differences in DG or CA3 neurons. Decreased level of 5-hmC immunoreactivity in cells of the DG and glial cells of the CA3 (-16.1%, p = 0.042 and -34.2%, p = 0.011 respectively).
Condliffe D. et al, 2014[30]	UK, n = 21, 78.18 ± 2.02 years, M and W	13	Cortical and cerebellar tissue	Age and gender	Inverse association	Significant decrease in 5-hmC in AD compared to controls (EC p<0.001, CER p = 0.0476). No differences found in 5-mC levels between AD and controls, nor between brain regions. No estimates given.
Lashley T. et al, 2014[31]	UK, n = 26, 71.8 ± 4.2 years, M and W	12	Brain tissue (entorhinal cortex and cerebellum)		No association	No significant differences detected between AD and control cases in either 5mC or 5hmC staining (both in immuno-histochemical analysis and ELISA).
Coppieters N. et al, 2014[33]	New Zealand, n = 58, 75.35 ± 9.2 years, M and W	29	Cortical tissue: In middle frontal gyrus (MFG) and middle temporal gyrus (MTG)	Age at death and post-mortem delay matched	Positive association	Significant increase in global levels (integrated intensity per cell) of 5mC (p = 0.0304) and 5hmC (p = 0.0016) in MFG of AD cases compared to controls. Significant increase of 5mC (p<0.0001) and 5hmC (p<0.0001) each in MTG of AD cases compared to controls.
Rao J.S. et al, 2012[34]	USA, n = 20, 70.4 ± 2.4, Gender not specified	10	Post-mortem frontal cortext (Brodmann area 9)		Positive association	The AD brains showed significant increases in global DNA methylation compared to age-matched controls.
Bednarska-Makaruk M. et al, 2016[32]	Poland, 194, 71.1 ± 7.56, M and W	53	PB	Age	No association	No significant differences detected between AD and control cases.
**5hmeC (5-hydroxymethylation)**						
Mastroeni D. et al, 2016[35]	USA, n = 12, 79–96, M and W	N = 6	Sub ventricular zone	Age	Positive association	There was an increase in DNA hydroxymethylation levels in AD compared to age-matched controls.
**LINE-1 methylation**						
Bollati V. et al, 2011[16]	Italy, n = 81, 71.2 ± 8.3 years, M and W	43	PB	Age and gender	Positive association	LINE-1 methylation was significantly increased in AD patients compared to controls (83,6% vs. 83,1 p = 0.04).
HernandezH. et al, 2014[36]	Columbia, n = 58, 76,2 ± 11.7 years, M and W	28	PBMCs	Age and gender	No association	No significant difference in median LINE-1 methylation levels between AD group and control group. There was also no difference between the groups when men and women were compared separately. There was also no difference seen when stratified for APOE-±4 carrier status.
**ALU**						
Bollati V. et al, 2011[16]	Italy, n = 81, 71.2 ± 8.3 years, M and W	43	PB	Age and gender	No difference	No difference.
**HpaII/MspI ratio**						
Shwob NG. et al, 1990[39]	Canada, n = 64, 45–92 years, M and W	44	Human post-mortem brain tissue (frontal cortex)		No difference	No difference in DNA methylation level between cases and controls (54.1 ± 2.26% vs. 52.9 ± 1.79%).
Basile AM. et. al,1997[37]	Italy		Lymphocytes		Positive association	DNA hypermethylation characterized the AD individuals.
**LUMA**						
DiFrancesco A. et al, 2015[38]	Italy, n = 81, 79.5 ±6.33 years, M and W	37	PBMCc		Positive association	Global DNA methylation levels were significantly increased in patients with LOAD compared to controls (p = 0.0122).
**H2**						
Anderson KW. et al, 2015[80]	USA, n = 16, 72–92.1 years old, M and F	6	Post-mortem frontal cortex		No difference	No difference in isoforms K/R99 or without K/R99
**H3**						
Zhang K. et al, 2012[79]	USA, n = 15, 54–101 years, M and W	11	Temporal lobe		Inverse association	Histone H3(H3K18/ K23) acetylation in AD cases was lower than in controls (six fold and p<0.02). This study also showed that SRM-based targeted proteomics, compared to western blot method and LC-MS/MS-TMT, showed higher throughput and therefore promises to be more suitable for clinical applications.
Rao JS. et al, 2012 [34]	USA, n = 20, 70.4 ± 2.4, Gender not specified	10	Post-mortem frontal cortex (Brodmann area 9)		Positive and no association	H3 phosphorylation was increased in AD brains compared to age-matched controls. No difference was observed in H3 acetylation.
Anderson KW. et al, 2015[80]	USA, n = 16, 72–92.1 years old, M and W	6	Post-mortem frontal cortex		No difference	K4- and K9-acetylated H3 did not show statistically significant changes between AD and control
Naryan PJ. et al, 2015[81]	New Zealand, n = 67, 75.4 ± 9.2, M and W	29	Post-mortem inferior temporal gyrus		Positive association	Acetyl histone H3 and acetyl histone H4 levels,as well as total histone H3 and total histone H4 protein levels, were significantly increased in post-mortemAlzheimer's disease brain tissue compared to age- and sex-matched neurologically normal control brain tissue. The increase in acetyl histone H3 and H4 was observed in Neuronal N immunopositive pyramidal neurons in Alzheimer's disease brain.
**H4**						
Anderson KW. et al, 2015[80]	USA, n = 16, 72–92.1 years old, M and W	6	Post-mortem frontal cortex		Positive and no difference	K8-, K12- and K16-acetylated H4 did not show statistically significant changes between AD and control. However, there was a 25% increase in K12- and K-16 acetylated H4.
Plagg B. et al, 2015[82]	Austria, n = 80, age and sex not defined	34	Monocytes		No difference	No difference in H4K12 acetylation was observed between AD patients and controls.
**PARKINSON’S DISEASE**						
**LINE-1 methylation**						
Nielsen SS. et al,2012[40]	USA (n = 693), 66.7 ± 9.5 years, M and W	292	PBMCs	Age, sex and smoking	No association	No association was observed between LINE-1 methylation and the presence of PD (p>0.40).
**Histone modifications**						
Gebremedhin KG. et al, 2016[84]	USA, n = 17, 71–87 years, M and W	9	Primary motor cortex		Positive and no difference	There was net increase in histone H3 acetylation due to increased H3K14 and H3K18 acetylation. There was a decrease in H3K9 acetylation. No between-groups difference was detected in H3K23 acetylation
Park G. et al, 2016[83]	USA, n = 10, 67.8–79.2 years, M and W	5	Postmortem midbrain tissues	Age and sex	Positive	Levels of histone acetylation (H2Ak5, H2Bk15, H3k9, and H4k5) are markedly higher in midbrain dopaminergic neurons of PD patients compared to those of their matched control individuals.

**Table 2:** Specific gene methylation in Alzheimer’s disease: gene and genome-wide approaches.

Author	Study design	Population/Age range/Follow-up	Cases	Tissue type	Methylation sites/methods	Adjustments	Main finding
**Candidate gene approach**							
An S. et al, 1994[135]	CCS/ Comparison of skin fibroblasts of AD and age/sex-matched controls	N = 4* Age and sex unspecified	N = 2	Skin fibroblasts	2-5A synthetase gene/Methylation-sensitive restriction enzymes (HpaII).		Hypo-methylation
Arosio B. et al, 2012[136]	CCS/Comparison of subjects with late onset AD (LOAD) and age-matched controls	Italy, n = 60, 79.7 ± 6.3 years, M and W	N = 32	PBMCs	*PIN1* gene promoter region/ bisulphite labelled RT-PCR		Hypo-methylation
Bajic V. et. al, 2014[137]	CCS/ Comparison of female AD patients and healthy age-matched controls	Serbia, n = 20, 68.1 ± 6.5 years, W	N = 10	PBMCs	Androgen receptor promoter (as a measure of X-inactivation pattern)/ MethySYBR Assay		Hyper-methylation
Banzhaf-Strathmann J. et al, 2013[53]	CCS/ Comparison between AD patients and age-matched neurologically healthy controls.	Multiple countries, n = 51, 70.5 ± 7.7 years, M and W.	N = 8	Human post-mortem brain tissue (frontal cortex)	*GRN* promoter/ Sequenom MassARRAY platform		No difference
Barrachina M. et. al, 2009[50]	CCS/ Comparison of AD (different stages) and controls.	European Brain Bank network (BrainNet Europe II), N = 70, 73.1 ± 10.1 years	N = 44	Human post-mortem brain tissue	CpG methylation in *MAPT*, *PSEN1*, *APP*, *UCHL1*/ SEQUENOM (Hamburg, Germany) MassArray System.Other: evaluation of effect of post-mortem delay on methylation analysis; comparison to other pathologies (FTD, PD etc.)		No difference
Brohede J.et al, 2010[51]	CCS	Sweden, n = 6, Five M, one W.	N = 6	Brain tissue (cortical and cerebellar).	12 CpG sites in the amyloid precursor protein gene (*APP*)/ bisulphite-PCR sequencing by 3100 Genetic analyzer		No difference
Chang L.et al, 2014[44]	CCS/ comparison of AD patients and age- and gender matched controls	China, n = 106, M and W.	N = 44	PB	*BDNF* promoter (4 CpG islands) / Pyromark Gold Q24 Reagents (Qiagen)	Age and gender matched	Hyper-methylation
D’addario C.et al, 2012[136]	CCS/ comparison of LOAD cases and age-matched controls	Italy, n = 66, 79.7 ± 7.8 years, M and W	N = 33	PBMCs	Methylation at fatty acid amide hydrolase (*FAAH*) gene promotor (18 CpG sites)/ methylation-specific primer real-time PCR.	Age matched	Hypo-methylation
DiFrancescoA. et al.,2013[138]	CCS/ comparison of LOAD subjects with age-matched controls	Italy, n = 55, 79.7 ± 6.34 years	N = 27	PBMCs	DNA methylation of *ALOX5* promoter	Age-matched controls	Hypo-methylation
Furuya T. et al, 2012[60]	CCS/ AD cases compared to healthy elderly and healthy young controls	Canada, Brain (n = 22), PB (n = 84), 62.9 ± 3.4 years, M and W	Brain: N = 12 Blood: N = 36	Brain (entorhinal cortex, auditory cortex, hippocampus) and PBMCs	*SORL1* and *SIRT1* gene methylation/ Sequenom EpiTYPER		No difference
Furuya T. et al, 2012[60]	CCS/ AD cases compared to healthy elderly and healthy young controls	Canada, Brain (n = 20), PB (n = 79), 63.5 ± 5.1 years, M and W	Brain: N = 10 Blood: N = 34	Brain (entorhinal cortex, auditory cortex, hippocampus) and PBMCs	*SNAP25* gene methylation/ Sequenom EpiTYPER	*ApoE4* status	No differences
Grosser C.et al, 2014[52]	CCS/ AD cases compared to controls	Netherlands, n = 10), 77.5 ± 13.3 years, M and W	N = 5	Brain tissue (middle temporal and superior frontal gyrus)	Methylation of *SST* and *SSTR4* promoter CpG islands (27 and 44 CpGs)/ Bisulphite RT-PCR sequencing	Age matched	No difference
Hou Y. et al,2013[49]	CCS/ AD cases compared to controls	China, n = 135, 78.4 ± 13.3 years, M and W	N = 63	PBMCs	CpG islands of *SIRT1* (SI and SII1/SII2) and amplifiable regions of *APP*, *ApoE4*, *PS1*, *PS2* and *Tau* / Bisulphite pyrosequencing (EZ DNA methylation Gold Kit)	Age, sex, scholarity and vascular disease matched	*SIRT1*: Hyper-methylation*APP*: Hypo-methylation*Apoe4*, *PS1*, *PS2* and *Tau*: No difference
Iwata A. et al, 2014[47]	CCS/ AD cases compared to controls	Japan, n = 158, 77.4 ± 6.1 years	N = 62	Brain tissue (cerebellum, anterior parietal lobe and inferior temporal lobe)	203 CpGs for *ACE*, *APOE*, *APP*, *BACE1*, *GSK3B*, *MAPT*, *PSEN1/* Bisulphite pyrosequencing by Pyromark Q24 analyzer (Qiagen)	Age-matched samples	Hypermethylation of CPGs in *APP*, *MAPT* and *GSK3B*.
Kaut O. et al, 2014[139]	CCS/ AD cases compared to controls	Germany, PB, n = 105, 69.7 ± 7.6 years.Cortical tissue, n = 8, 77.15 ± 10.0 years. M and W	N = 55 and n = 4	PBMCs and cortical tissue	TNF-α promoter. 10 CpGs analyzed by bisulphite sequencing PCR		Cortex: Hypo-methylationPBMC: No difference
Nagata T. et al, 2015[43]	CCS/ Comparison of AD patients with age-matched controls.	Japan, n = 40, 66.5 ± 5.09 years, M and W	N = 20	PBMCs	*BDNF* promoter 20 CpGs/ bisulfite sequencing		Hyper-methylation
Sanchez-Mut JV. et al, 2013[45]	CCS/ Comparison of AD patients with age and gender matched non-AD subjects.	eBrainNet Europe Bank / n = 40, 76,5 ± 2,5 years.	N = 20	Human post-mortem brain tissue (grey matter of frontal cortex)	*F2RL2*, *SORB3*, *SPNB4* and *TBX2AR*/ bisulfite pyrosequencing		*TBX2AR*, *SORBS3* and *SPTBN4*: Hyper-methylation*F2RL2*: No difference
Siegmund KD. et al, 2007[46]	CCS/ Comparison of AD patients with controls (including schizophrenic subjects).	USA, N = 58,60–104.3 years, M and W	N = 18	Human post-mortem brain tissue (temporal and frontal cortex)	50 loci related to central nervous system growth and development (*SORBS3*, *S100A2*, *LDLR*, *MYOD1*, *MGMT*, *LZTS1*, *GDNF*, *PYCARD*, *STK11*, *UIR*, *CRABP1*, *PLAGL1*, *DIRAS3*, *PGR*, *SERPINB5*, *NEUROD2*, *GAD1*, *RNR1*, *ALU*, *TFAP2A*, *MINT1*, *CDKN2A*, *NTF3*, *SASH1*, *PAX8*, *SYK*, *NEUROD1*, *PSEN1*, *ALU*, *GABRA2*, *DRD2*, *LTBR4*, *ALU*, *HOXA1*, *CALCA*, *DNAJC15*, *SMAD3*, *CDX1*, *SCGB3A1*, *MT1A*, *TNFRSF25*, *MTHFR*, *MGMT*, *FAM127A*, *AR*, *LPHN2*, *ALU*, *RASSF1*, *BDNF*)/ bisulfite pyrosequencing.		*SORB3*: Hyper-methylation*S100A2*: Hypo-methylationOther genes: No difference
Silva PN. et al, 2014[59]	CCS/ Comparison of AD patients with non-AD controls.	Canada, n = 79, 75.7 ± 8.2 years, M and W	N = 46	PB and human post-mortem brain tissue	*HSPA8* and *HSPA9*, 22 and 34 CpGs respectively/ Sequenom EpiTyper MassARRAY		No difference overall, but differentially methylated CpG sites
Silva PNO. et al, 2008[54]	CCS/ Comparison of AD patients with age matched non-AD controls and young controls.	Brazil, n = 145, 57.2 ± 4.9 years, M and W	N = 45	PB	*SIRT3*, *SMARCA5*, *HTERT* and *CHD1* gene/ bisulfite pyrosequencing		*HTERT*: Hyper-methylation*SIRT3*, *SMARCA5* and *CHD1*: No difference
Wang SC. et al, 2008[140]	CCS/ Comparison of late onset-AD patients with geographical location, ethnicity, age and sex matched non-AD controls	Germany, n = 34, 80.6 ± 9.4 years, M and W	N = 24	Human post-mortem brain tissue (prefrontal gyrus frontalis superior) and blood lymphocytes	12 AD’s susceptibility loci (*HTATIP*, *MTHRF*, *DNMT1*, *TFAM*, *SIN3A*, *NCSTN*, *BACE1*, *APP*, *PSEN1 APH1B* and *APOE*)/ bisulfite pyrosequencing (MALDI-TOF mass spectrometry analysis)		No difference.
Wang Y. et al, 2014[62]	CCS/ Comparison of AD patients with age and sex matched non-AD controls.	China, n = 50, 75.4 ± 9.1 (60–90) years,M and W	N = 25	Blood lymphocytes.	DR4 gene promoter, 2 CpG islands (9 and 13 CpG sites each)/ Bisulfite sequencing		Hypo-methylation
West RL. et al, 1995[48]	CCS/ Comparison of female AD patients with age-matched controls.	USA, n = 3, 83, 74 and 81 years, W	N = 12	Human post-mortem brain tissue (Brodmann’s area 38)	Amyolid precursor protein (*APP*) and superoxide dismutase (*SOD-1*) genes/ Methylation-sensitive restriction enzymes (HpaII).		*APP*: Hypo-methylation*SOD-1*: No difference
Rao JS. et al, 2012[34]	CCS/ Comparison of AD patients with age-matched controls.	USA, n = 20, 70.4 ± 2.4 years, Gender not specified	N = 10	Human post-mortem brain tissue (Brodmann’s area 9)	Promoter of *COX-2*, *BDNF*, *NF-kβ*, *CREB*, *12-LOX*, p450 epoxygenase, synaptophysin and debrin-like genes/ Methylation-sensitive restriction enzymes		*COX-2* and *NF-kβ*: Hypo-methylation*BDNF*, synaptophysin and *CREB*: Hyper-methylation*12-LOX*, debrin-like protein or p450 epoxygenase: No difference
Yu L. et al, 2015[61]	CCS/ Comparison of AD patients with non-AD controls.	USA, n = 740, 88 ± 6.7 years, M and W	N = 447	Human post-mortem brain tissue (gray matter)	28 reported AD loci/ Infinum HumanMethylation 450: Illumina)	Age, sex, batch, bisulfite conversion efficacy, macroscopic and microscopic infarcts and cortical Lewy bodies	Results vary per CpG sites
Carboni L. et al, 2015[55]	CCS/ Comparison of AD patients with non-AD controls.	Italy, n = 39, 75 ± 7 years, M	N = 20	Peripheral blood	Promoter of *BDNF*, *SIRT1* and *PSEN1* / Bisulfite sequencing		No difference
Celarain N. et al, 2016[141]	CCS/ Comparison of AD patients with non-AD controls.	Spain, n = 42, 19 to 98 years, M and W	N = 30	Frozen postmortem hippocampussamples	*TREM2* transcription start site (TSS)-associated region / Bisulfite sequencing		Hypermethylation
Coppedè F. et al, 2016[56]	CCS/ Comparison of late onset-AD (LOAD) patients with non-AD controls.	Italy, n = 111, 77.1 ± 8.8 years, M and W	N = 56	PB	Genes involved in major DNA repair pathways: *OGG1*, *PARP1*, *MRE11A*, *BRCA1*, *MLH1*,and *MGMT*/ effectivePCR based methylation-sensitive high-resolution melting (MS-HRM) technique	Age, gender and multiple comparison	No difference
Ferri E. et al, 2016[142]	CCS/ Comparison of AD patients with non-AD controls.	Italy, n = 283, 79.4 ± 0.5 years, M and W	N = 176	PBMCs	Pin1 gene promoter, 5 CpG sites / Bisulfite sequencing	Age and gender	No difference
Foraker J. et al, 2015[143]	CCS/ Comparison of AD patients with non-AD controls.	USA, n = 25, 83.6 ± 9 years, M and W	N = 15	Postmortem brain, cerebellum, hippocampus, frontal lobe	*APOE*, 76 CpG sites/ Bisulfite sequencing	Age, sex, disease status, *APOE*genotype, CpG site, and tissue type, as well as allsecond-order interactions involving tissue	Hypermethylated
Ji H. et al, 2015[144]	CCS/ Comparison of sporadic AD patients with non-AD controls.	China, n = 106, 80.4 ± 8.4 years, M and W	N = 48	PB	Promoter *OPRK1*, 3 CpG sites/ Bisulphite pyrosequencing	History of smoking, diabetes and hypertension	Hypermethylated
Ma SL. et al, 2016[58]	CCS/ Comparison of AD patients with non-AD controls.	China, n = 260, 81.3 ± 7.0 years, W	N = 80	PB	*CTSB*, *CTSD*, *DDT*, *TSC1*, *NRD1*, *UQCRC1 and NDUFA6* / Bisulphite pyrosequencing		Hypermethylated and no difference
Tannorella P. et al, 2015[57]	CCS/ Comparison of sporadic AD patients with non-AD controls.	Italy, n = 223, 76.6 ± 8.2 years, M and W	N = 120	PB	The promoter/5-UTR regions of *PSEN1*, *BACE1*, *MTHFR*, *DNMT1*, *DNMT3A*, and *DNMT3B* / Bisulphite pyrosequencing	Age at sampling, gender, homocysteine, folate, vitamin B12 and batch	No difference
Mendioroz M. et al, 2016[145]	CCS/ Comparison of AD patients with non-AD controls.	Spain, n = 42, age and sex not defined	N = 30	Hippocampus	*CRTC1* gene / Bisulphite pyrosequencing		Hypomethylation
**Genome-wide approach**							
Bakulski K. et al, 2012[71]	CCS/Comparison of subjects with LOAD and age- and gender-matched controls	USA, n = 24, 79.8 years (range 69–95)(13 additional matched pairs for the population validation phase, 78.2 years (range 61–95)),M and W	N = 12/N = 13	Human post-mortem frontal cortex tissue	Genome-wide DNA methylation profile. 27,578 CpG sites spanning 14,475 genes/ Infinium HumanMethylation27 BeadArray (Illumina).Gene-specific DNA methylation/bisulfite-pyrosequencing on the Qiagen Pyromark MD (Valencia, CA).Other: gene expression, protein quantification	Age and gender	948 CpG sites representing 918 unique genes potentially associated with LOAD disease status (p<0.05). Across these sites the mean methylation difference between cases and controls is 2.9%.Hypermethylation in AD cases of molecular function and biological processes associated with transcription (e.g. RNA polymerase II transcription factor activity).Hypomethylation in AD cases of functions relating to membrane transport and protein metabolism.The CpG site in the promoter of the Transmembrane Protein 59 (*TMEM59*) gene is 7.3% hypomethylated in AD cases.
De Jager PL. et al, 2014[72]	CCS/ comparison of participants in a prospective cohort study, with post-mortem diagnosis of AD.	USA, n = 708, M and W	60.8% (N = 430) of subjects met a pathological diagnosis of AD.	Cortical brain tissue	Methylation at 425,848 discrete CpG dinucleotides in 708 subjects (Illumina HumanMethylation beadset).Other: Identification of genes near the associated CpGs.		137 CpGs were found to be associated with the burden of neuritic amyloid plaques (NP) (p<1.20x 10^-7). When corrected for the proportion of neurons and possible measurement artifacts, 71 CpG associations remained.22 of the NP-associated CpG s were also associated with AD at a genome-wide level of significance, and all displayed at least (p<0.001) some evidence of association with AD. Associated methylated regions included *ABCA7* and *BIN1* genes, which are known AD susceptibility regions.
Fernandez AF. et al, 2012[146]	CS/ whole genome methylation “fingerprint” including normal tissues, oncogenic tissues, and non-cancerous disease tissues (such as AD and DLB)	Europe, Asia and North America, n = 1628, M and W	N = 11	Brain tissue and PBMCs	1322 CpG sites/ Golden Gate DNA methylation BeadArray (Illumina), Pyromark Q24 (Qiagen)		No significant difference was found between brain samples from AD patients and normal tissues.
Humphries C. et Al, 2015[73]	CCS/ AD cases compared to healthy controls and diseased controls (DLB)	USA, n = 30, 77.0 ± 4.5 years	N = 8	Brain tissue	DNA methylation analysis including 5,147 CpG sites on 465 genes/ Illumina Infinium HumanMethylation 450 beadchip		1,106 CpG sites differed in LOAD-associated methylation network genes between LOAD and control subjects (p<0.05). Hypomethylation was observed in LOAD subjects in 87.3% of these CpG sites.
Sanchez-Mut JV. et al, 2014[74]	CCS/ Comparison of AD patients with non-AD subjects.	Spain,Discovery set: n = 20, 79.7 ± 1.9 years. Replication set: n = 50, 71.7 ± 2.1 years, M and W	Discover set, n = 15.Replication set, n = 25	Human post-mortem brain tissue (grey matter, Brodmann area 9)	Illumina 27K array assay and bisulfite pyrosequencing		In the discovery set, four CpG methylation probes corresponding to 3 individual genes showed a significant difference between AD-cases and controls (P<0.05); two hypermethylated CpGs in dual specificity phosphatase 22 (*DUSP22*), 1 CpG in claudin 15 (*CLDN15*) and and 1 CpG in quiescin Q66 sulfhydryl oxidase 1 (*QSCN6*). In the replication set, the hypermethylation of *DUSP22* was confirmed.
Bernstein AI. et al, 2016[75]	Comparison of AD with control cases	USA, n = 11, 78–91 years, M and W (both discovery and replication set)	N = 6	Human post-mortem brain tissue (frontal cortex)	5-methylcytosine and 5-hydroxymethylcytosine (5hmC)		There were 325 genes containing differentially hydroxymethylated loci (DhMLs) in bothdiscovery and replication datasets. These are enriched for pathways involved in neuron projection development andneurogenesis.
Watson CT. et al, 2016[76]	CCS/ Comparison of AD patients with non-AD subjects.	USA, n = 68, 66–95 years, M and W	N = 34	Bulk tissue samples from the superior temporagyrus	461,272 autosomal CpGs / HumanMethylation450 platform	AOD,gender, race, array/batch, and neuronal/glial cell composition.	There were 479 differentially methylated regions (DMR) ((increased in AD; hyper-DMRs = 321, hypo-DMRs = 158), with relevant roles in neuron function and development, as well as cellular metabolism. Top DMRs were close to following genes: *MOV10L1*, *B3GALT4*, *DUSP6*, *TBX15*, *HLA-J*, *ZNRD1-AS1*, *PRDM16*, *ELOVL1*, *RIBC2*, *SMC1B*, *KLK7*, *TRIM6*, *FBRSL1*, *VAX2*, *CDH23*, *KIF25*, *NRG2*, *RNF39*, *CMYA5*, *TNXB*, *NAV2*, *TAP2*, *ZNF177*, *FLOT1*.

**Table 3:** Specific gene methylation in Parkinson’s disease: gene and genome-wide approaches.

Author	Study design	Population/Age range/Follow-up	Cases	Tissue type	Methylation sites/methods	Adjustments	Main finding
**Candidate gene approach**							
Ai SX. et al, 2014[62]	CCS/ Comparison between PD patients and neurologically healthy controls	China, n = 195, 61.8 ± 9.7 years, M and W	N = 100	PBMCs	23 CpG sites in the *SNCA* gene/ Bisulphite pyrosequencing (Epitect Bisulfite Kite, Qiagen).Other: genotyping of Rep1 (polymorphic dinucleotide repeat upstream of SNCA), rt-PCR of *SNCA*	Age, gender and origin matched	Hypo-methylation
Banzhaf-Strathmann J. et al, 2013[53]	CCS/ Comparison between PD patients and age-matched neurologically healthy controls.	Multiple countries, n = 51, 70.5 ± 7.7 years, M and W.	N = 8	Human post-mortem brain tissue (frontal cortex)	*GRN* promoter/ Sequenom MassARRAY platform		No difference
Cai M. et al, 2011[67]	CCS/ Comparison between PD patients (with and without heterozygous Parkin gene mutations) and neurologically healthy controls	China, n = 44, M and W	N = 34 (17 with heterozygous Parkin gene mutations and 17 without)	PBMCs	33 CpG sites in the Parkin gene promoter region/ Bisulphite sequencing (EZ DNA Methylation Kit, Zymo Research).	Age, gender and ethnicity matched	No difference
Coupland KG. et al, 2014[70]	CS	Australia,n = 1442 leukocyte samples + 109 PD brain tissue DNA samples.	N = 386	Leukocyte DNA and brain tissue DNA	Six CpGs in the *MAPT* gene. Methylation assessed by bisulphite pyrosequencing (PyroMark Q24, Qiagen).Other: in vitro *MAPT* promoter methylation assay and Vitamin E assay	In leukocytes, adjustment for (amongst others) smoking, L-dopa medication, gender, age, *MAPT* diplotype.In brain tissue (cerebellum), adjustment for age, sex and *MAPT* diplotype	Hyper-methylation in the cerebellum. Hypo-methylation in the putamen.
Jowaed A. et. al, 2010[19]	CCS/ Comparison between PD patients and neurologically healthy controls	Germany, n = 26, 77.5 ± 3.8 years, M and W	N = 12	Brain tissue (substantia nigra pars compacta (SNpc) and cortex and putamen)	Bisulphite sequencing of 23 CpG sites in the *SNCA* gene		Hypo-methylation
Song Y. et al, 2014[66]	CCS/ Comparison of PD patients with age, gender, ethnicity and area of residence matched controls.	China, n = 100, 72.3 ± 7.6 years, M and W	N = 50	Blood leucocytes	α-synuclein gene (*SNCA*), 13 CpGs/ bisulfite pyrosequencing		No difference
Lin Q. et al, 2012[68]	CCS/ Comparison of PD patients with age and gender non-PD controls.	China, n = 386, 66.2 ± 3.4 years, M and W	N = 206	Blood leucocytes	Clock genes (*PER1*, *PER2*, *CRY1*, *CRY2*, *CLOCK*, *NPAS2* and *BMAL1*)/ bisulfite pyrosequencing		*NAPS2*: Hypo-methylation.Other genes: No difference
Tan Y. et al, 2014[63]	CCS/ Comparison of PD patients with age and gender matched non-PD controls.	China, n = 200, 65.2 ± 0.12 years, M and W	N = 100	Blood leucocytes	α-synuclein gene (*SNCA*) (2CpGs islands, 30 CpGs) and *LRRK2* (1 CpG island, 34 CpGs) promoter/ bisulfite Specific PCR-based and bisulfite Specific Cloning-based		Hypo-methylation
Villar-Menendez I. et al, 2014[147]	CCS/ Comparison of PD patients with age matched non-PD controls.	Spain, n = 19, 24–85 years, M and W	N = 7	Human post-mortem brain tissue (putamen)	*ADORA2A*, 3 CpG island, 108 CpG sites/ Sequenom EpiTyper MassARRAY		Hypo-methylation
Nielsen SS. et al, 2015[148]	CCS/ Comparison of PD cases with non-PD controls.	USA, n = 201, 25–65 years, M	N = 49	WB	*NOS2*, 3CpGs/ bisulfite pyrosequencing	Age, examiner and experimental plate	Hypo-methylation
Matsumoto L. et al, 2010[64]	CCS/ Comparison of PD cases with non-PD controls.	Japan, n = 20, 57–87 years, M and W	N = 11	Human post-mortem brain tissue (anterior cingulate, putamen and substantia nigra)	α-synuclein gene (*SNCA*), CpG-2 / bisulfite sequencing		Hypo-methylation
Tan Y. et al, 2016[69]	CCS/ Comparison of PD cases with non-PD controls.	China, n = 80, 62.5 ± 7.8 years, M and W	N = 40	Peripheral bloodleukocytes	*DJ-1*, 2 CpGs / bisulfite sequencing	Age	No difference
Su X. et al, 2015[149]	CCS/ Comparison of PD cases with non-PD controls.	USA, n = 20, 78.3 ± 8.1 years, M and W	N = 10	Substantia nigra	Peroxisome proliferator-activated receptor gamma coactivator−1 α (PGC−1α)/ bisulfite sequencing	Age	Hypermethylated
Schmitt I. et al, 2015[65]	CCS/ Comparison of PD cases with non-PD controls.	Germany, n = 975, 64.6 ± 9.6 years, M and W	N = 490	PB	α-synuclein	Not clear	Hypomethylated
**Genome-wide approach**							
Kaut O. et al, 2012[77]	Case control study/ Comparison between PD patients and neurologically healthy controls	Germany, n = 18, 78.6 ± 10.1 years, M and W	N = 6	Brain tissue (cortex and putamen)	Genome-wide methylation. 17,500 individual CpG sites from 14,495 genes.(EZ DNA Methylation Gold Kit (Zymo Research) and Illumina Human-Methylation27 BeadChip).		In both cortex and putamen of PD patients, *CYP2E1* was hypomethylated (Mean β-value: 0.37 ±0.27 (control) vs. 0.07±0.06 (PD), p = 0.04 and 0.48±0.17 (control) vs 0.07±0.01 (PD), p = 0.0005 respectively). This difference remained when the analysis was stratified by gender.In the cortex of PD patients, the gene *PPP4R2* was hypomethylated (0.50±0.30 (control) vs. 0.32±0.05 (PD), p = 0.02) in comparison to controls. In the putamen of PD patients, the gene *MGC 3207* was hypomethylated when compare to controls(0.47 ±0.22 (control) vs. 0.16±0.13 (PD), p = 0.02). In the putamen of PD patients, *DEFA1* and *CHFR* were hypermethylated.
Masliah E. et al, 2013[78]	Genome-wide DNA methylation Case control study/ Comparison of PD cases with age matched non-PD controls.	USA (n = 11), M and W	N = 5	Human post-mortem brain tissue (frontal cortex) and PBL	485386 CpG/ HumanMethylation 450k BeadChip (Illumina # WG-314-1003)		2908 CpG—174 genes (317 hypermethylated-84 genes and 2591 hypomethylated -90 genes) in the brain and 3897 CpG– 233 genes (476 hypermethylated-127 genes and 3421 hypomethylated-106 genes) in the blood of PD cases were differentially methylated compared to controls. 30% (124/407) of the total autosomal annotated genes differentially methylated presented concordant changes in methylation between blood and brain (63 loci with increased methylation and 61 with decreased methylation), suggesting that a number of methylation changes in PD is shared between brain and blood, positioning these 124 genes that co-varied among tissues as candidates for biomarker discovery. Top 30 loci: hypermethylated in PD: *KCTD5*, *VAV2*, *MOG*, *TRIM10*, *HLA-DQA1*, *ARHGEF10*, *GFPT2*, *HLA-DRB5*, *TMEM9*, *MRI1*, *MAPT*, *HLA-DRB6*, *MAPT*, *HLA-DRB6*, *LASS3*, *GSTTP2* and *GSTTP1*; Hypomethylated in PD: *DNAJA3*, *JAKMIP3*, *FRK*, *LRRC27*, *DMBX1*, *LGALS7*, *FOXK1*, *APBA1*, *MAG12*, *APBA1*, *SLC25A24*, *GSTT1*, *MYOM2*, *MIR886*, *TUBA3E* and *TMCO3*. Gene ontology analysis showed that same functional groups were affected in brain and blood, with cell communication and cellular and metabolic processes being the more populated clusters, and including genes related to apoptosis, a molecular pathway largely implicated in PD.Overall methylation patterns of the brain and blood were similar, with more than 80% of the sites reported as differentially methylated being hypomethylated. While there were no differences between brain and blood in CpGs clustering in low-methylated fraction, there were more CpGs in the high-methylated fraction in PD blood in comparison to control subject’s blood and also to PD brains (P<0.001). CpG neighbourhood context analysis and genomic location distribution was comparable between brain and blood samples and showed that loci with decreased methylation were more likely to locate at CG islands and associated with promoter regions including TSS1500, TSS200 and 1^st^ exon sites; while CpG sites located further away from islands (open sea) and at the gene bodies were more likely to present increased methylation.

### Global DNA Methylation

Global methylation refers to the overall level of methylcytosine in the genome, expressed as percentage of total cytosine. Many of the methylation sites within the genome are found in repeat sequences and transposable elements, such as Alu and long-interspersed nuclear element (LINE-1). They correlate with the total genomic methylation content. Measurements of methylation of the repetitive elements in the genome are used as a surrogate measurement for the overall methylation of the genome. Some studies quantified global DNA methylation by calculating the amount of methylated cytosines in the sample (5 mc) relative to global cytidine (5mC + dC) in a positive control. Other methods to assess global genomic DNA methylation (e.g., Luminometric Methylation Assay (LUMA) and the [^3^H]-methyl acceptance based method) are primarily based on the digestion of genomic DNA by restriction enzymes HpaII, MspI and Dpn I.

Thirteen studies examined the association between global DNA methylation and AD (**Table 1**). Eight studies assessed DNA methylation in brain tissue and the rest of the studies assessed it in blood cells. Seven studies assessed global DNA methylation as a percentage of 5-methylcytosine in samples from brain. Of these seven studies, three studies [28–30] found lower levels of methylation in AD cases compared to controls, two studies [31, 32] found no difference, and two other studies [33, 34] reported higher levels of methylation in AD subjects. One study [35] reported an increase in DNA 5-hydroxymethylation levels in AD compared to age-matched controls. One study [36] assessed global DNA methylation in LINE-1 elements in blood and showed no difference between AD patients and healthy controls. One study [16] examined global DNA methylation in both LINE-1 and Alu elements. It reported no difference in global DNA methylation levels in Alu elements, and reported higher levels of methylation in LINE-1 elements in blood cells of AD compared to healthy controls. Three studies used other methods to assess global DNA methylation: two studies [37, 38] reported DNA hypermethylation in AD individuals whereas one study [39] showed no difference in global DNA methylation between AD cases and controls.

There was only one study that examined the association between global DNA methylation at LINE-1 elements in blood and PD. The study showed no association [40] (**Table 1**).

### Gene Specific DNA Methylation

DNA methylation, the addition of a methyl group to the 5’ position of cytosine in a dinucleotide CpG site, is an important mechanism in gene expression regulation. The direction of association between DNA methylation and gene expression depends on where within the gene sequence the methylation occurs. DNA methylation in the promoter region of the gene down-regulates its expression whereas higher methylation in the gene body may promote the expression of the gene [41]. However, in most instances DNA methylation represses gene expression. It is thought that methylation of DNA either directly prevents binding of DNA transcription factors, or it recruits proteins that bind to methylated DNA. Recruiting proteins may prevent transcription by influencing chromatin structure by histone modification [41, 42]. The effects of DNA methylation allow for the evaluation of gene function by comparing individuals who have the methylated or unmethylated versions of a gene. These methylation patterns can be studied both in a candidate gene approach or a genome-wide approach.

#### 1. Candidate Gene Studies

Thirty-four studies examined methylation sites in or near known candidate genes for AD susceptibility in relation to AD (Table 2). The 34 studies showed that AD cases, compared to controls, have a higher degree of methylation of *OPRK1*, *UQCRC1*, *AR*, *BDNF* and *HTERT* in blood cells, *BDNF*, synaptophysin gene, *CREB* promoters, *APOE*, *TREM 2*, *TBX2AR*, *SORBS3* and *SPTBN4* in the brain, and lower methylation levels of 2-5a-synthetase gene in skin fibroblasts, *PIN1*, *FAAH*, *ALOX5* and DR4 gene in blood cells, *TNFA*, *COX-2*, NF-kβ gene and *S100A2* and *CRTC1* in the brain tissue. The most consistently reported epigenetic associations with AD were that of methylation at *BDNF* [34, 43, 44] in both blood and brain tissue, and at *SORBS3* [45, 46] in the frontal cortex, which were reported in three and two studies respectively. However, one study [46] did not find a difference in DNA methylation of *BDNF* gene in AD brain compared to healthy controls. The most studied epigenetic mark in relation to AD was the methylation pattern of the *APP* gene. The *APP* gene was investigated in five studies: three studies (two studies using brain samples [47, 48] and one study using blood cells [49]) showed hypomethylation of *APP* in AD cases compared to controls. Alternatively, two studies [50, 51] showed no difference in DNA methylation of *APP* in brain tissue between AD and healthy controls. Fourteen studies found no difference or clear pattern in methylation of the following genes: *12-LOX* [34], debrin-like protein gene [34], p450 epoxygenase gene [34], *MAPT*, *PSEN1*, *UCHL1*, *SST* [52], *SSTR4* [52], *F2RL2* [45], *SOD-1* [48] and *GRN* [53] in brain tissue; *PS1* [49], *PS2* [49] and tau1 [49], *SMARCA 5* [54], *CHD1* [54], *BDNF* [55], *SIRT1* [55], *PSEN1* [55{Tannorella, 2015 #2823], genes involved in DNA repair [56], genes involved in homocysteine pathway [57], *CTSB* [58], *CTSD* [58], *DDT* [58], *TSC1* [58], *NRD1* [58] and *NDUFA6* [58] in blood cells; *HSPA8* [59], *HSPA9* [59], *ApoE4* [47, 49], *SNAP25* [60], *SORL 1*, *SIRT1* and *SIRT3* [49, 54, 60] in both blood cells and brain tissue (Table 2). However, 7 studies showed differences in methylation patterns of CpG sites (within same gene some CpG sites were hypomethylated and some others were hypermethylated, in AD cases) examined at the following genes: *SORL1* [61], *ABCA7* [61], *SLC2A4* [61], *BIN1* [61], *HSPA8* [59], *HSPA9* [59], *DR4* gene [62], *BDNF4* [43, 44], *SIRT1* [49], *APP* [47], *MAPT* [47] and *GSK3B* [47].

There were 13 studies that examined methylation sites in or near known candidate genes for PD susceptibility (**Table 3**). Overall the studies looking at PD found lower levels of methylation of *NAPS2* and *NOS2* in blood cells and of *ADORA2A* in the brain tissue of PD cases, and higher levels of methylation of PGC−1α gene in brain tissue of PD patients. Six studies examined the methylation pattern of α-synuclein gene (*SNCA*) in blood and brain tissue in relation to PD: 5 studies [19, 62–65] showed significantly decreased levels of methylation in PD patients compared to controls whereas 1 study [66] found a non-significant decrease in PD subjects. Four studies [53, 67–69] did not show any difference in DNA methylation of the following genes: Parkin gene, *DJ-1*, *PER1*, *PER2*, *CRY1*, *CRY2*, *CLOCK* and *BMAL1* in blood cells and of *GRN* in brain tissue. One study [70] examined DNA methylation of the *MAPT* gene in blood cells and different areas of the brain and showed that the association between DNA methylation of *MAPT* and presence of PD differ by the tissue examined.

#### 2. Genome-wide analysis

Six studies looked for differentially methylated sites associated with AD in brain tissue; 1 study also looked in both brain tissue and blood cells (Table 2). Up to 1106 CpG sites were reported to be differentially methylated in the brains of AD cases compared to individuals without AD. One study [71] found 948 CpGs representing 918 unique genes in the frontal cortex were associated with late onset-AD status. In AD cases, there was mainly hypermethylation of genes related to molecular and biological processes involved in transcription, and hypomethylation of genes related to membrane transport and protein metabolism (e.g. *TMEM59*). One study reported that out of 137 CpGs in cortical brain tissue found to relate with the burden of natriuretic amyloid plaques (NP), 22 were also associated with the presence of AD [72]. Another study [73] reported 1106 CpGs to be differentially methylated in late onset-AD subjects compared to healthy controls and that 87,3% of the CpG sites were hypomethylated. Among the CpGs found to differ in methylation frequency between AD patients and healthy controls in the initial analysis, only the hypermethylation of *DUSP22* gene in AD cases could be confirmed in the replication set [74]. Two other studies [75, 76] reported that differentially methylated regions in the brain tissue of AD patients were related to genes involved in neurogenesis, neuronal projection development and regulation of neuron differentiation, as well as β-amyloid and tau metabolism.

Two studies conducted an epigenome-wide association study approach for PD. One study reported hypomethylation of *CYP2E1*, *PPP4R2* and *MGC3207* and hypermethylation of *DEFA1* and *CHFR* in the putamen and cortex of PD cases compared to controls [77]. Another study [78] found 2908 CpGs (317 hypermethylated and 2591 hypomethylated) in the brain tissue and 2897 CpGs (476 hypermethylated and 3421 hypomethylated) in the blood cells of PD patients to be differentially methylated compared to controls. The study found that 30% of the differentially methylated sites presented concordant changes in methylation between blood and brain. The identified genes were enriched for genes (known from genome-wide association studies) with epigenetic changes in biological pathways relevant to PD-development, such as cell communication and apoptosis (**Table 3**).

### Histone Modifications and Neurodegenerative disorders

Five studies [34, 79–82] examined histone modification in relation to AD. There were no consistent findings on the role of H3 or H4 acetylation in AD (**Table 1**). However, one of the studies [34] showed increased H3 phosphorylation in AD brains compared to age-matched controls (**Table 1**).

There were two studies [83, 84] examining the role of histone modifications in PD. They mainly showed an increase in levels of histone acetylation in PD patients.

## Discussion

We have systematically reviewed the current knowledge about epigenetic associations with Alzheimer’s disease (AD) and Parkinson’s disease (PD). There is some evidence that DNA methylation may be related to the risk of neurological disease. Among gene-specific studies, DNA methylation at 24 genes was found to be associated with AD, while 7 genes were differentially methylated in PD.

The present review finds inconsistent associations between global DNA methylation and AD. These results are in line with previous studies showing contradictory results when studying the relationship between global DNA methylation and other health outcomes, including cardiovascular disease and diabetes [85–91]. The use of different methods for assessing global DNA methylation, including the 5-methylcytosine ratio and the methylation of LINE-1 and Alu repeat elements, may account for some of these differences. LINE-1 and Alu repeat elements are used as a measure of global DNA methylation due to their ubiquitous presence in the genome. However, as they may have different functions, the resulting differences in methylation may explain some of the conflicting results [92]. DNA methylation at Alu is about one-third to one-fourth of methylation at LINE-1. The difference may suggest that epigenetic changes at LINE-1 and Alu measure different traits [92]. Global DNA methylation assessed by LUMA modestly correlates with LINE-1 methylation, suggesting that the differences in the reported results may come from the assay used to assess global DNA methylation [93]. Furthermore, as different tissue types (brain tissue or peripheral blood samples) are assessed between studies, tissue-specific DNA methylation patterns may partially explain the heterogeneous findings. Even within studies performed on brain tissue, samples are obtained from different areas of the brain, including cortical, cerebellar, and hippocampal tissue. This difference may limit comparability of the results as specific brain regions comprise different cell populations (astrocytes, neurons, microglia, oligodendrocytes). Furthermore, the same methylation pattern, depending on its position toward coding gene, can have different effects [41, 94]. Therefore, global DNA methylation provides an oversimplified assessment of epigenetic dysregulation, as it neither quantitatively nor qualitatively acknowledges the co-existence of hypo- and hypermethylation within a gene or distinct genes within the same cell.

In our review, several genes were found to be differentially methylated in brain tissue or peripheral blood of AD patients when compared to controls. In particular, brain derived neurotrophic factor (*BDNF*) and *SORBS3* were each found in two different studies to be significantly more methylated in AD patients than in controls. These results parallel previous studies showing an association between *BDNF* hypermethylation in blood and depression, depressive symptoms and antidepressants response [95]. Similarly, previous studies have reported hypermethylation of *BDNF* and of its receptor (Tropomyosin-Related Kinase B) in brains of individuals who have committed suicide [96, 97]. BDNF is a secretory protein with neuroprotective effects [98] which has been shown to be associated with neurodegenerative diseases, including AD, PD and Huntington’s disease [99]. *BDNF* was shown to be hypermethylated in the peripheral blood of AD patients compared to controls, indicative of decreased expression of *BDNF*. This is consistent with findings in brain tissue of patients diagnosed postmortem with AD [34] and with other studies showing that *BDNF* promoter methylation is related to *BDNF* mRNA expression [97]. As BDNF is able to cross the blood-brain barrier [100], DNA methylation in the peripheral tissue may exert effects on neuronal tissue and vice versa, highlighting the potential utility of peripheral *BDNF* methylation as a biomarker for AD. This is supported by the overlap of epigenetic changes in both AD-brain tissue and peripheral blood reported in this review. *SORBS3* is involved in neuronal signaling [101] and regulation of gene expression [102], and was found in two studies to be hypermethylated in the frontal cortex of AD patients. However, its role in the pathogenesis of AD and whether methylation of *SORBS3* is consistent across tissue types remains to be investigated.

Also, genes of proteins implicated in AD pathogenesis, such as CREB, were differentially methylated in PD, but the evidence is too limited to draw a firm conclusion. AD is associated with a reduction of CREB activation. CREB is a histone acetyltransferase that functions as a co-activator that catalyzes histone acetylation, causing a decrease in the transcription of memory-associated genes, and therefore, leading to memory impairment [103]. Treatment targeting the transcription machinery interacting with CREB during memory formation has been suggested to be a useful strategy for treating AD [103]. Furthermore, genes of proteins such as death receptor 4 (DR4) and NF-ĸB are involved in processes that may play a role in the pathogenesis of AD such as apopotosis and/or inflammation. DR4 and NF-ĸB genes were reported to be differentially methylated in AD cases [104, 105]. DR4 might impair the apoptotic signal transduction and may cause apoptosis of brain cells[104]. Polymorphisms of the DR4 gene have been shown to influence susceptibility to AD [104]. NF-ĸB activation is a common feature of many neurodegenerative diseases, particularly of AD [105]. Activation of NF-ĸB leads to the expression of a large variety of pro-inflammatory molecules such as cytokines and chemokines, which could be in part responsible for the neurotoxicity seen in AD [105]. The interaction of methylation of these genes with molecular pathways and how this affects risk of AD remains to be elucidated.

In PD patients, *SNCA* was consistently found to be hypomethylated in both peripheral blood cells and brain tissue. Known to be a causative gene of familial PD [106], the overexpression of *SNCA* in sporadic PD cases [107–109] suggests a role in the pathogenesis of sporadic PD as well. The finding that *SNCA* is similarly hypomethylated in both peripheral blood and in brain tissues is in line with previous studies and indicates it may be useful as a biomarker in sporadic PD.

Also, several other genes involved in the pathogenesis of PD were reported to be differentially methylated in PD cases, including *NOS2* (hypomethylated), *ADORA2A* (hypomethylated), and *CYP2E1* (hypomethylated). *NOS2*, the gene coding for inducible nitric oxide synthase (iNOS) is primarily regulated at the transcriptional level, at least partially via DNA methylation [110]. Hypomethylation of CpG sites in the 5′ promoter region of the gene might increase iNOS expression [110]. Increased iNOS expression in turn promotes inflammation and may lead to PD [111]. In line with this evidence, a selective iNOS inhibitor, GW274150 ([2-[(1-iminoethyl) amino] ethyl]-L-homocysteine) has been reported to have a neuroprotective effect in a model of PD [112]. *ADORA2A* is the gene coding for adenosine A2A receptor (A2AR), which is highly expressed in the striatum. *ADORA2A* polymorphisms have been inversely associated with PD risk [113]. Also, A2AR antagonists are effective in relieving parkinsonian motor symptoms and have been suggested as potential new drugs for PD treatment [114]. *CYP2E1* codes for Cytochrome P450 2E1, a member of the Cytochrome P450 enzyme family, which represent a major part of the cellular defense against xenobiotic exposure and have been implicated in PD pathophysiology since the mid-1980s [115]. Decreased methylation of *CYP2E1* is related to increased expression of CYP2E1 messenger RNA in PD patients [77]. Enhanced CYP2E1 activity has been suggested to contribute to dopaminergic neurodegeneration in PD [115, 116].

This review demonstrated that while epigenetic changes in AD and PD patients have been investigated via global methylation and gene-specific methylation studies, findings are lacking regarding histone modification. Histone modifications are another epigenetic mark that play a pivotal role in the epigenetic regulation of transcription and other functions in cells, including neurons [117]. Posttranslational histone modifications interfere with the transcriptional program inducing long-lasting phenotypic changes in neural plasticity including learning and memory [118, 119]. Many enzymes are involved in the regulation of histones including processes such as acetylation, methylation, phosphorylation, sumoylation and ubiquitination, which may play important roles in the pathogenesis of ND [120]. Histone deacetylases (HDACs) has been reported to be active in these processes. Valproic acid, an inhibitor of HDACs, demonstrates neuroprotection against rotenone in a rat model of PD [121]. Also in AD and PD animal models, histone acetylation has been linked to neurodegeneration [120, 122]. One study in Huntington’s disease patients found that most of the identified histone modifications in the brain are associated with genes that have known roles in neuronal signaling [123]. Those findings suggest that histone modifications may be a relevant form of epigenetic change in patients of neurological diseases. Therefore, much information may still be gained from histone modification studies in AD or PD patients.

The strengths and limitations of the findings from this review merit careful consideration. The present report involves data from nearly 11,453 individuals. It is the first systematic review on the subject that has critically appraised the literature following an *a priori* designed protocol with clearly defined inclusion and exclusion criteria. Using a systemic search in medical databases, few reviews evaluating the role of epigenetics marks in AD and PD were found [124–126]. Existing reviews were all narrative reviews (not performed systematically). Narrative reviews do not involve a systemic search and they are often focused on a subset of studies in the chosen area based on availability of the author selection. Therefore, they are more likely to experience selection bias. A number of limitations, however, need to be considered. The majority of studies included in our systematic review are cross-sectional assessments, making it difficult to draw conclusions on causality. Also, studies investigating epigenetic dysregulation in neurological diseases suffer from small sample size, the consequences of which include reduced statistical power and increased false discovery rates. In addition, although most of the epigenetic studies included in this review adjusted for age and sex and sampled from an ethnically homogenous population, a number of analyses are lacking adjustment for lifestyle and environmental factors. Factors including smoking and alcohol consumption are important risk factors for neurological disorders and can alter epigenetic mechanisms. Furthermore, when assessing epigenetic modifications, studies used different techniques, which may produce heterogeneous results. Also, genetically, AD and PD are usually divided into familial cases with Mendelian inheritance and sporadic cases with no familial aggregation [127]. The sporadic form is more complex and likely results from a combination of genetic and environmental influences. Therefore, examining whether epigenetic marks may have different role in the etiology of AD and PD types would be interesting [127]. Most of the studies included in this review used post-mortem brain tissue, which can help to provide several insights about the nature of epigenetic medications in relation to neurodegenerative diseases, but can also present several limitations. Using post-mortem brain is problematic with respect to temporality between exposure and outcome[128]. Second, untangling real effects from confounders (such as medications) can be challenging. Lastly, death often involves acidosis, which can alter genetic material, increasing the likelihood of misclassifying epigenetic modification and increasing the chances of spurious findings [129, 130].

## Conclusion

Overall, the findings from this review indicate that there are significant epigenetic differences between patients with neurodegenerative diseases and healthy individuals. Furthermore, candidate gene studies have shown that some genes known to play a role in maintenance and function of neurological tissues are differentially methylated in diseased individuals. In addition, a number of these genes, such as *BDNF* in AD patients and *SNCA* in PD patients, are similarly methylated in blood and brain tissue. Along the same lines, Epigenetic Wide Association Studies show that differentially methylated sites in neurological disorders present concordant changes in methylation between blood and brain. These data suggest that studies in peripheral blood can provide valuable information on the neuronal epigenetic changes and their consequences on cell function. Therefore, methylation profiling in peripheral blood to identify neurological disorders-related methylated regions has a high potential clinical utility. It may allow clinicians to identify high-risk individuals who may benefit from preventive and therapeutic interventions. However, due to the mostly cross-sectional design of included studies and lack of replication in the case of new findings, there remain many questions about the temporal relation between epigenetic modifications and neurological diseases, as well as the significance of the findings in disease pathology. Also, given the reversible nature of epigenetic aberrations, targeting the epigenome can be a novel preventive strategy and treatment for AD and PD. There is evidence showing that methyl donors such as folate and vitamin B12 may affect DNA methylation and the risk for several neurodegenerative conditions, including AD and PD [131, 132]. Studies from animal studies show that histone deacetylase inhibitors lowers Aβ levels and improves learning and memory in a mouse model of Alzheimer's disease. Those findings provide support that histone deacetylase inhibitors may serve as a novel therapeutic strategy for AD [133]. Epigenetic therapy has been shown to successfully reverse several epigenetics marks and disease symptoms and have been approved by the FDA for use in cancer [134]. Therefore, studies in larger cohorts with longitudinal design may help to close the gap on identifying epigenetic changes that have clinical significance and could lead to strategies for intervention in neurological diseases.

## Supporting Information

S1 File PRISMA 2009 checklist (DOCX)

S2 File MOOSE checklist (DOCX)

S3 File Search strategy (DOCX)

S4 File Thirty-two full-text excluded articles (DOCX)

S5 File Newcastle-Ottowa Quality Assessment Scale checklist (DOCX)

S6 File List of frequently used abbreviations (DOCX)
