# Supplementary material for: The Role of DNA Methylation and Histone Modifications in Neurodegenerative Diseases: A Systematic Review
Source: PLoS One. 2016 Dec 14;11(12):e0167201. doi: 10.1371/journal.pone.0167201 (PMC5156363; doi:10.1371/journal.pone.0167201)
Supplement: S2 File — (DOCX) [file pone.0167201.s002.docx]

**S2: Moose checklist**

| **Criteria** | | **Brief description of how the criteria were handled in the meta-analysis** |
| --- | --- | --- |
| **Reporting of background should include** | |  |
| √ | Problem definition | Epigenetic modifications of the genome, such as DNA methylation and histone modifications, have been reported to play a role in processes underlying neurodegenerative diseases (ND), including Alzheimer’s disease and Parkinson’s disease. To date, however, little work has been done to systematically appraise the current evidence for the role of DNA methylation and histone modifications on the risk of ND. |
| √ | Hypothesis statement | Epigenetic modifications (DNA methylation and histone modifications) are associated with the risk of ND. |
| √ | Description of study outcomes | Alzheimer’s disease and Parkinson’s disease |
| √ | Type of exposure or intervention used | Global DNA methylation, gene-specific DNA methylation and histone modifications. |
| √ | Type of study designs used | We included cross-sectional studies, case control studies and prospective studies. |
| √ | Study population | Adult individuals from the general population |
| **Reporting of search strategy should include** | |  |
| √ | Qualifications of searchers | The credentials of the investigators are indicated in the authors list. |
| √ | Search strategy, including time period included in the synthesis and keywords | Search strategy and time periods are detailed in page 5 of the manuscript and in eAppendix 3. |
| √ | Databases and registries searched | Embase.com, Medline (Ovid), Web-of-Science, Scopus, PubMed, Cinahl (EBSCOhost), Cochrane Central, ProQuest, Lilacs, Scielo and Google Scholar. |
| √ | Search software used, name and version, including special features | We did not employ any search software. EndNote was used to merge retrieved citations and eliminate duplications. |
| √ | Use of hand searching | We hand-searched bibliographies of retrieved papers and relevant reviews for additional references. |
| √ | List of citations located and those excluded, including justifications | Details of the literature search process are outlined in the flow chart. Citations for the included studies are enclosed in the table 1, 2 and 3. The citation list for excluded studies is available upon request. |
| √ | Method of addressing articles published in languages other than English | We placed no restrictions on language. All identified studies were in English. |
| √ | Method of handling abstracts and unpublished studies | No unpublished studies were identified |
| √ | Description of any contact with authors | None |
| **Reporting of methods should include** | |  |
| √ | Description of relevance or appropriateness of studies assembled for assessing the hypothesis to be tested | Detailed inclusion and exclusion criteria are described in the Methods section. |
| √ | Rationale for the selection and coding of data | Data extracted from each of the studies were relevant to the population characteristics, study design, exposure, outcome, and possible effect modifiers of the association |
| √ | Assessment of confounding | We assessed confounding by ranking individual studies on the basis of different adjustment levels. |
| √ | Assessment of study quality, including blinding of quality assessors; stratification or regression on possible predictors of study results | Study quality was assessed using the Newcastle-Ottawa Scale, a semi-quantitative scale designed to evaluate the quality of nonrandomized studies. |
| √ | Assessment of heterogeneity | We did not perform any test of heterogeneity. |
| √ | Description of statistical methods in sufficient detail to be replicated | Description of methods of systematic review and assessment of publication bias are detailed in the methods. |
| √ | Provision of appropriate tables and graphics | We included 3 main tables. |
| **Reporting of results should include** | |  |
| √ | Graph summarizing individual study estimates and overall estimate | NA |
| √ | Table giving descriptive information for each study included | Table 1 and Table 2 and Table 3 |
| √ | Results of sensitivity testing | NA |
| √ | Indication of statistical uncertainty of findings | 95% confidence intervals were presented with all summary estimates. |
| **Reporting of discussion should include** | |  |
| √ | Quantitative assessment of bias | Newcastle-Ottawa scale was used to assess bias. |
| √ | Justification for exclusion | We excluded studies that used different exposure or outcome assessment for the comparison groups. |
| √ | Assessment of quality of included studies | We discussed the results of the Newcastle-Ottawa scale, and potential reasons for the observed heterogeneity. |
| **Reporting of conclusions should include** | |  |
| √ | Consideration of alternative explanations for observed results | We discussed that potential unmeasured confounders may have caused residual confounding. Additionally, our findings could have been over-estimated somewhat due to preferential publication of extreme findings. |
| √ | Generalization of the conclusions | The generalizability of our findings has been enhanced by the involvement of data from 11,453 participants. |
| √ | Guidelines for future research | We recommend future studies evaluate the impact of epigenetic marks in ND. |
| √ | Disclosure of funding source | No separate funding was necessary for the undertaking of this systematic review. |
