# Supplementary material for: The Role of DNA Methylation and Histone Modifications in Neurodegenerative Diseases: A Systematic Review
Source: PLoS One. 2016 Dec 14;11(12):e0167201. doi: 10.1371/journal.pone.0167201 (PMC5156363; doi:10.1371/journal.pone.0167201)
Supplement: S3 File — (DOCX) [file pone.0167201.s003.docx]

**S3** Search strategy

**Embase.com**

(epigenetics/exp OR 'DNA methylation'/exp OR 'histone modification'/exp OR 's adenosylmethionine'/exp OR 'CpG island'/exp OR 'histone deacetylase inhibitor'/exp OR 'histone acetyltransferase'/exp OR (((histone* OR dna OR 'long interspersed') NEAR/3 (acetyla* OR demethylat* OR deacetyla* OR acetyltransferase* OR methylat* OR phosphorylat* OR ubiquitinat* OR modif*)) OR 's adenosylmethionine' OR cpg OR epigenetic* OR epigenomic* OR neuroepigenetic* OR neuroepigenomic* OR (HDAC NEAR/3 inhibitor*)):ab,ti) AND (dementia/exp OR 'Parkinson disease'/exp OR 'degenerative disease'/exp OR 'nerve degeneration'/exp OR (dement* OR Alzheimer* OR huntington* OR rett OR prion OR Parkinson* OR ((neuro* OR nerve*) NEAR/3 degenerat*) OR neurodegenerat*):ab,ti) NOT ([animals]/lim NOT [humans]/lim) NOT ([Conference Abstract]/lim OR [Letter]/lim OR [Note]/lim OR [Editorial]/lim)

**Medline (OvidSP)**

(Epigenomics/ OR "DNA methylation"/ OR "Histone Code"/ OR "S-Adenosylmethionine"/ OR "CpG Islands"/ OR exp "Histone Deacetylase Inhibitors"/ OR exp "Histone Acetyltransferases"/ OR (((histone* OR dna OR "long interspersed") ADJ3 (acetyla* OR demethylat* OR deacetyla* OR acetyltransferase* OR methylat* OR phosphorylat* OR ubiquitinat* OR modif*)) OR "s adenosylmethionine" OR cpg OR epigenetic* OR epigenomic* OR (HDAC ADJ3 inhibitor*)).ab,ti.) AND (exp dementia/ OR "Parkinson Disease"/ OR exp "Neurodegenerative Diseases"/ OR exp "Nerve Degeneration"/ OR (dement* OR Alzheimer* OR huntington* OR rett OR prion OR Parkinson* OR ((neuro* OR nerve*) ADJ3 degenerat*) OR neurodegenerat*).ab,ti.) NOT (exp animals/ NOT humans/) NOT (letter OR news OR comment OR editorial OR congresses OR abstracts).pt.

**PsycINFO (OvidSP)**

(Epigenetics/ OR (((histone* OR dna OR "long interspersed") ADJ3 (acetyla* OR demethylat* OR deacetyla* OR acetyltransferase* OR methylat* OR phosphorylat* OR ubiquitinat* OR modif*)) OR "s adenosylmethionine" OR cpg OR epigenetic* OR epigenomic* OR (HDAC ADJ3 inhibitor*)).ab,ti.) AND (exp dementia/ OR "Parkinson's Disease"/ OR exp "Neurodegenerative Diseases"/ OR exp "Neurodegeneration"/ OR (dement* OR Alzheimer* OR huntington* OR rett OR prion OR Parkinson* OR ((neuro* OR nerve*) ADJ3 degenerat*) OR neurodegenerat*).ab,ti.) NOT (exp animals/ NOT humans/) NOT (letter OR news OR comment OR editorial OR congresses OR abstracts).pt.

**Cinahl (ebsco)**  (MH Epigenomics+ OR MH "DNA methylation+" OR MH "S-Adenosylmethionine+" OR (((histone* OR dna OR "long interspersed") N3 (acetyla* OR demethylat* OR deacetyla* OR acetyltransferase* OR methylat* OR phosphorylat* OR ubiquitinat* OR modif*)) OR "s adenosylmethionine" OR cpg OR epigenetic* OR epigenomic* OR (HDAC N3 inhibitor*))) AND (MH dementia+ OR MH "Parkinson Disease+" OR MH "Neurodegenerative Diseases+" OR MH "Nerve Degeneration+" OR (dement* OR Alzheimer* OR huntington* OR rett OR prion OR Parkinson* OR ((neuro* OR nerve*) N3 degenerat*) OR neurodegenerat*)) NOT (MH animals+ NOT MH humans+) NOT PT (letter OR news OR comment OR editorial OR congresses OR abstracts)

**Cochrane**

((((histone* OR dna OR 'long interspersed') NEAR/3 (acetyla* OR demethylat* OR deacetyla* OR acetyltransferase* OR methylat* OR phosphorylat* OR ubiquitinat* OR modif*)) OR 's adenosylmethionine' OR cpg OR epigenetic* OR epigenomic* OR (HDAC NEAR/3 inhibitor*)):ab,ti) AND ((dement* OR Alzheimer* OR huntington* OR rett OR prion OR Parkinson* OR ((neuro* OR nerve*) NEAR/3 degenerat*) OR neurodegenerat*):ab,ti)

**Web-of-science**

TS=(((((histone* OR dna OR "long interspersed") NEAR/3 (acetyla* OR demethylat* OR deacetyla* OR acetyltransferase* OR methylat* OR phosphorylat* OR ubiquitinat* OR modif*)) OR "s adenosylmethionine" OR cpg OR epigenetic* OR epigenomic* OR (HDAC NEAR/3 inhibitor*))) AND ((dement* OR Alzheimer* OR huntington* OR Parkinson* OR ((neuro* OR nerve*) NEAR/3 degenerat*) OR neurodegenerat*)) NOT ((animal OR mouse OR mice OR rat OR rats OR murine OR primate* OR monkey*) NOT (human* OR patient*))) AND dt=(article)

**Scopus**

TITLE-ABS-KEY(((((histone* OR dna OR "long interspersed") W/3 (acetyla* OR demethylat* OR deacetyla* OR acetyltransferase* OR methylat* OR phosphorylat* OR ubiquitinat* OR modif*)) OR "s adenosylmethionine" OR cpg OR epigenetic* OR epigenomic* OR (HDAC W/3 inhibitor*))) AND ((dement* OR Alzheimer* OR huntington* OR Parkinson* OR ((neuro* OR nerve*) W3 degenerat*) OR neurodegenerat*)) AND NOT ((animal OR mouse OR mice OR rat OR rats OR murine OR primate* OR monkey*) AND NOT (human* OR patient*))) AND doctype(ar)

**PubMed publisher**

(Epigenomics[mh] OR "DNA methylation"[mh] OR "Histone Code"[mh] OR "S-Adenosylmethionine"[mh] OR "CpG Islands"[mh] OR "Histone Deacetylase Inhibitors"[mh] OR "Histone Acetyltransferases"[mh] OR (((histone*[tiab] OR dna OR "long interspersed") AND (acetyla*[tiab] OR demethylat*[tiab] OR deacetyla*[tiab] OR acetyltransferase*[tiab] OR methylat*[tiab] OR phosphorylat*[tiab] OR ubiquitinat*[tiab] OR modif*[tiab])) OR "s adenosylmethionine" OR cpg OR epigenetic*[tiab] OR epigenomic*[tiab] OR (HDAC AND inhibitor*[tiab]))) AND (dementia[mh] OR "Parkinson Disease"[mh] OR (dement*[tiab] OR Alzheimer*[tiab] OR huntington*[tiab] OR rett OR prion OR Parkinson*[tiab] OR neuro degenerat*[tiab] OR nerve degenerat*[tiab] OR neurodegenerat*[tiab])) NOT (animals[mh] NOT humans[mh]) NOT (letter[pt] OR news[pt] OR comment[pt] OR editorial[pt] OR congresses[pt] OR abstracts[pt]) AND publisher[sb]

**Google scholar**

"histone|dna acetylation|demethylation|deacetylation|acetyltransferase|methylation|phosphorylation|modification"|cpg|epigenetic|epigenetics|epigenomic|epigenomics dementia|Alzheimer|huntington|Parkinson| degenerative|neurodegenerative|neurodegeneration

**Proquest**

(ab(epigenetics OR epigenomics) OR ti(epigenetics OR epigenomics)) AND (ab(dementia OR Alzheimer OR huntington OR Parkinson OR neurodegeneration OR neurodegenerative OR "nerve degeneration") OR ti(dementia OR Alzheimer OR huntington OR Parkinson OR neurodegeneration OR neurodegenerative OR "nerve degeneration"))

**Lilacs**

**Scielo**

(epigenetics OR epigenomics) AND (dementia OR Alzheimer OR huntington OR Parkinson OR neurodegeneration OR neurodegenerative OR "nerve degeneration")
