# Supplementary material for: The Role of DNA Methylation and Histone Modifications in Neurodegenerative Diseases: A Systematic Review
Source: PLoS One. 2016 Dec 14;11(12):e0167201. doi: 10.1371/journal.pone.0167201 (PMC5156363; doi:10.1371/journal.pone.0167201)
Supplement: S4 File — (DOCX) [file pone.0167201.s004.docx]

**S4** List of excluded articles.

***No appropriate exposure***

1. Drake, J., et al., 4-Hydroxynonenal oxidatively modifies histones: Implications for Alzheimer's disease. Neurosci Lett, 2004. 356(3): p. 155-158.

2. Graff, J., et al., An epigenetic blockade of cognitive functions in the neurodegenerating brain. Nature, 2012. 483(7388): p. 222-226.

3. Guan, J.Z., et al., Effect of vitamin E administration on the elevated oxygen stress and the telomeric and subtelomeric status in Alzheimer's disease. Gerontology, 2012. 58(1): p. 62-69.

4. Guan, J.Z., et al., The Subtelomere of Short Telomeres is Hypermethylated in Alzheimer's Disease. Aging dis., 2012. 3(2): p. 164-170.

5. Guan, J.Z., et al., Analysis of telomere length and subtelomeric methylation of circulating leukocytes in women with Alzheimer's disease. Aging Clin Exp Res, 2013. 25(1): p. 17-23.

6. Mulder, C., et al., The transmethylation cycle in the brain of Alzheimer patients. Neurosci Lett, 2005. 386(2): p. 69-71.

7. Sontag, E., et al., Downregulation of protein phosphatase 2A carboxyl methylation and methyltransferase may contribute to Alzheimer disease pathogenesis. J. Neuropathol. Exp. Neurol., 2004. 63(10): p. 1080-1091.

8. Linnebank, M., et al., S-adenosylmethionine is decreased in the cerebrospinal fluid of patients with Alzheimer's disease. Neurodegenerative Dis, 2010. 7(6): p. 373-378.

9. Speranca, M.A., et al., Can the rDNA methylation pattern be used as a marker for Alzheimer's disease? Alzheimer's Dementia, 2008. 4(6): p. 438-442.

10. Obeid, R., et al., Methylation status and neurodegenerative markers in Parkinson disease. Clin Chem, 2009. 55(10): p. 1852-1860.

11. Maeda, T., et al., Aging-associated alteration of telomere length and subtelomeric status in female patients with Parkinson's disease. J Neurogenet, 2012. 26(2): p. 245-251.

12. Maeda, T., et al., Aging-associated alteration of subtelomeric methylation in Parkinson's disease. J Gerontol Ser A Biol Sci Med Sci, 2009. 64(9): p. 949-955.

13. Xie, T., et al., Is X-linked methyl-CpG binding protein 2 a new target for the treatment of Parkinson's disease? Neural Regen Res, 2013. 8(21): p. 1948-1957.

14. Choong, C.J., et al., A novel histone deacetylase 1 and 2 isoform-specific inhibitor alleviates experimental Parkinson's disease. Neurobiol Aging, 2016. 37: p. 103-116.

15. Alsadany, M.A., et al., Histone deacetylases enzyme, copper, and IL-8 levels in patients with Alzheimer's disease. Am J Alzheimer's Dis Other Dem, 2013. 28(1): p. 54-61.

16. Bayram, A., et al., Decreased HDAC1 gene expression in patients with alzheimernulls disease. Int J Hum Gen, 2014. 14(3-4): p. 177-182.

17. Cook, C., et al., Acetylation of the KXGS motifs in tau is a critical determinant in modulation of tau aggregation and clearance. Hum Mol Genet, 2014. 23(1): p. 104-116.

***Not the appropriate outcome***

1. Cook, C., et al., Loss of HDAC6, a novel CHIP substrate, alleviates abnormal tau accumulation. Hum Mol Genet, 2012. 21(13): p. 2936-2945.

2. Ding, H., P.J. Dolan, and G.V.W. Johnson, Histone deacetylase 6 interacts with the microtubule-associated protein tau. J Neurochem, 2008. 106(5): p. 2119-2130.

3. Drzewinska, J., A. Walczak-Drzewiecka, and M. Ratajewski, Identification and analysis of the promoter region of the human DHCR24 gene: Involvement of DNA methylation and histone acetylation. Mol Biol Rep, 2011. 38(2): p. 1091-1101.

4. De Boni, L., et al., Next-generation sequencing reveals regional differences of the (alpha)-synuclein methylation state independent of Lewy body disease. NeuroMol Med, 2011. 13(4): p. 310-320.

5. Hou, Y., et al., Changes in methylation patterns of multiple genes from peripheral blood leucocytes of Alzheimer's disease patients. Acta Neuropsychiatr, 2013. 25(2): p. 66-76.

6. Dompierre, J.P., et al., Histone deacetylase 6 inhibition compensates for the transport deficit in Huntington's disease by increasing tubulin acetylation. J Neurosci, 2007. 27(13): p. 3571-3583.

7. Hoshino, M., et al., Histone deacetylase activity is retained in primary neurons expressing mutant huntingtin protein. J Neurochem, 2003. 87(1): p. 257-267.

8. Ryu, H., et al., ESET/SETDB1 gene expression and histone H3 (K9) trimethylation in Huntington's disease. Proc Natl Acad Sci U S A, 2006. 103(50): p. 19176-19181.

9. Hu, Y., et al., Transcriptional modulator H2A histone family, member Y (H2AFY) marks Huntington disease activity in man and mouse. Proc Natl Acad Sci U S A, 2011. 108(41): p. 17141-17146.

10. Vashishtha, M., et al., Targeting H3K4 trimethylation in Huntington disease. Proc Natl Acad Sci U S A, 2013. 110(32): p. E3027-E3036.

11. Yeh, H.H., et al., Histone deacetylase class II and acetylated core histone immunohistochemistry in human brains with Huntington's disease. Brain Res, 2013. 1504: p. 16-24.

12. Galimberti, D., et al., Progranulin gene (GRN) promoter methylation is increased in patients with sporadic frontotemporal lobar degeneration. Neurol Sci, 2013. 34(6): p. 899-903.

13. Banzhaf-Strathmann, J., et al., Promoter DNA methylation regulates progranulin expression and is altered in FTLD. Acta Neuropathol Commun, 2013. 1(1): p. 16.

***Animal model and/or no relevant data***

1. Hao, Y., et al., Promoter characterization and genomic organization of the human X11beta gene APBA2. Neuroreport, 2012. 23(3): p. 146-151.
