# Supplementary material for: The Role of DNA Methylation and Histone Modifications in Neurodegenerative Diseases: A Systematic Review
Source: PLoS One. 2016 Dec 14;11(12):e0167201. doi: 10.1371/journal.pone.0167201 (PMC5156363; doi:10.1371/journal.pone.0167201)
Supplement: S5 File — (DOCX) [file pone.0167201.s005.docx]

**S5.** Newcastle-Ottowa Quality Assessment Scale checklist

| *Alzheimer’s disease* | | *Parkinson’s disease* | |
| --- | --- | --- | --- |
| *Author (year)* | *Score* | *Author (year)* | *Score* |
| Thomas (2013) | 6 | Ai (2014) | 9 |
| An (1994) | 3 | Banzhaf-Strathman (2013)* | 4 |
| Arosio (2012) | 8 | Cai (2011) | 7 |
| Bajic (2014) | 7 | Coupland (2014) | 6 |
| Bakulski (2012) | 8 | Jowaed (2010) | 6 |
| Bai (2015) | 7 | Kaut (2012) | 6 |
| Barrachina (2009) | 6 | Lin (2012) | 5 |
| Basile (1997) |  | Matsumoto (2010) | 4 |
| Masliah (2013) | 4 |  |  |
| Bollati (2011) | 8 | Nielsen (2012) | 8 |
| Brohede (2010) | 4 | Song (2014) | 6 |
| Chang (2014) | 8 | Tam (2014) | 4 |
| Chouliaras (2013) | 6 | Villar-Mendez (2014) | 5 |
| Condliffe (2014) | 5 | Anderson (2015) | 2 |
| Coppieters (2014) | 8 | Bednarska-Makaruk (2016) | 7 |
| D’Addario (2012) | 8 | Bernstein (2016) | 6 |
| De Jager (2014) | 6 | Carboni (2015) | 5 |
| Di Francesco (2015)* | 9 | Celarain (2016) | 5 |
| Di Francesco (2013)* | 9 | Coppedè (2016) | 7 |
| Fernandez (2012) | 3 | Ferri (2016) | 6 |
| Furuya (2012)* | 6 | Foraker (2015) | 6 |
| Furuya (2012)* | 5 | Gebremedhin (2016) | 6 |
| Grosser (2014) | 7 | Ji (2015) | 5 |
| Hernandez (2014) | 8 | Ma (2016) | 7 |
| Hou (2013) | 8 | Watson (2016) | 6 |
| Humphries (2015) | 7 | Tannorella (2015) | 7 |
| Iwata (2014) | 4 | Tan (2016) | 5 |
| Kaut (2014) | 3 | Su (2015) | 6 |
| Lashley (2014) | 7 | Schmitt (2015) | 5 |
| Mastroeni (2010) | 5 | Mendioroz (2016) | 5 |
| Nagata (2015) | 5 | Mastroeni (2016) | 5 |
| Sanchez-Mut (2014) | 5 | Narayan (2015) | 5 |
| Sanchez-Mut (2013) | 5 | Park (2016) | 5 |
| Shwob (1990) | 5 | Plagg (2015) | 6 |
| Siegmund (2007) | 4 |  |  |
| Silva (2014) | 5 |  |  |
| Silva 2008 | 4 |  |  |
| Wang (2008) | 4 |  |  |
| Wang (2014) | 5 |  |  |
| West (1995) | 4 |  |  |
| Yu (2015) | 9 |  |  |
| Zhang (2012) | 4 |  |  |

*Articles are based on same study
