# Supplementary material for: The Role of DNA Methylation and Histone Modifications in Neurodegenerative Diseases: A Systematic Review
Source: PLoS One. 2016 Dec 14;11(12):e0167201. doi: 10.1371/journal.pone.0167201 (PMC5156363; doi:10.1371/journal.pone.0167201)
Supplement: S6 File — (DOCX) [file pone.0167201.s006.docx]

**S6** List of frequently used abbreviations

| AD | Alzheimer's disease |
| --- | --- |
| *APP* | Amyloid precursor protein gene |
| *BDNF* | Brain derived neurotrophic factor |
| DNA | Deoxyribonucleic acid |
| HDAC | Histone deacetylase |
| HpaII | DNA restriction enzyme |
| LINE-1 | Long-interspersed nuclear element |
| LUMA | Luminometric Methylation Assay |
| MspI | DNA restriction enzyme |
| ND | Neurodegenerative diseases |
| NP | Neuritic amyloid plaques |
| PB | peripheral blood |
| PBL | peripheral blood leukocytes |
| PBMC | peripheral blood mononuclear cells |
| PD | Parkinson's disease |
| *SNCA* | α-synuclein gene |
| *SORBS3* | Vinexin gene |
| TNF-α | Tumor necrosis factor-α |
